# Exploratory investigation of dose calculation and tumor-feeding artery identification using dual CBCT in selective internal radiation therapy with Yttrium-90 microspheres

**DOI:** 10.3389/fonc.2025.1655769

**Published:** 2025-10-23

**Authors:** Zhaoxiong Guo, Yuchan Liang, Tingfeng Li, Ao Li, Kangshun Zhu, Yongjian Guo, Wenxin Wang, Wensou Huang

**Affiliations:** ^1^ Minimally Invasive Interventional Radiology, The Second Affiliated Hospital of Guangzhou Medical University, Guangzhou, China; ^2^ Guangdong Engineering Technology Research Center of Interventional Oncology and Precision Drug Delivery, Guangzhou, China; ^3^ Department of Nuclear Medicine, The Second Affiliated Hospital, Guangzhou Medical University, Guangzhou, China; ^4^ Philips Healthcare, Shanghai, China

**Keywords:** cone beam computed tomography (CBCT), Yttrium-90 (^90^Y), interventional oncology, selective internal radiation therapy (SIRT), CTA

## Abstract

**AIM:**

To explore the preliminary application of dual cone-beam CT (CBCT) for dose calculation and tumor-feeding arteries identification in ^90^Y-SIRT planning.

**Materials and Methods:**

A retrospective study analyzed 27 patients with unresectable primary/metastatic liver tumors eligible for ^90^Y-SIRT. Prior to angiography, dual CBCT and ^99^mTc-MAA injection, each patient underwent CTA scan. Tumor volume (TV) and liver lobe volume (LLV) were measured from CTA and dual CBCT images (TVcta *vs* TVcbct and LLVcta *vs* LLVcbct). Liver perfusion volume (LPV) was derived from ^99^mTc-MAA mapping and dual CBCT (LPVmma *vs* LPVcbct). Additionally, analyze the differences between an average calculated ^90^Y dosage derived from TVcbct and LPVcbct, and dosage calculated using TVcbct combined with LPVmma, against the mean clinically administered (Radioactivity). The Paired Wilcoxon test was applied to evaluate differences between these parameters throughout the study.

**Results:**

There were no significant differences in liver tumor and perfusion volume measurements (p-values of 0.792 and 0.084, respectively). There was a significant difference in LVcbct compare to LVcta (2083.88 ± 744.64 *vs* 2187.86 ± 807.28 cm³, p = 0.024), which may be due to differences in contrast agent delivery. No significant differences were found among the three methods of calculated ^90^Y dosage(TVcbct + LPVcbct, TVcbct +LPVmma, radioactivity)were (1.819 ± 1.241, 1.806 ± 1.240, 1.805 ± 1.236)(all P>0.05).

**Conclusion:**

Dual CBCT is a reliable alternative to the conventional method, while offering real-time procedural advantages for feeder artery identification and catheter positioning during ^90^Y-SIRT.

## 1 Background

In 2022, Selective Internal Radiation Therapy (SIRT) with Yttrium-90 microspheres, also known as ^90^Y radioembolization, was officially introduced in China for treating unresectable Hepatocellular Carcinoma (HCC) and metastatic liver cancer patients (1). In June of the same year, the first clinical consensus on Yttrium-90 (^90^Y) microspheres for SIRT in Asia was released. This consensus provided crucial guidelines for the clinical management of ^90^Y microspheres in liver tumors such as HCC and Colorectal Liver Metastases (CRLM) in China (2). By selectively delivering radioactive ^90^Y microspheres through the tumor’s blood supply arteries, the therapy precisely targets tumor cells, minimizing systemic side effects and significantly prolonging patient survival compared to non-selective internal radiation therapy, while protecting normal liver tissue (3–15).

It is recommended to perform angiography with selective injection of Technetium-99m labeled macroaggregated albumin (^99m^Tc-MAA) at the anticipated SIRT site to identify tumor-feeding vessels and quantify the liver-lung shunt ratio, simulating ^90^Y procedure conditions. Clinical consensus also recommends meticulous preoperative imaging planning during the ^99m^Tc simulation for ^90^Y procedures. Optimal catheter placement and safe administration of the radioactive material should be ensured by referencing images such as preoperative computed tomography angiography (CTA), cone-beam CT (if available), digital subtraction angiography (DSA), and catheter position alignment (11).

The prediction of traditional partition model relies on preoperative CTA or MRI images (5, 11), typically starting with measuring tumor volume using four-phase liver CTA images in China. However, due to the dependence of CTA scans on peripheral vein injection of contrast agents, both tumor and surrounding tissues are enhanced, resulting in relatively lower contrast agent concentration in the tumor region. This limited enhancement effect in the liver and tumor areas poses challenges in accurately assessing liver lobe volume and tumor volumes. Additionally, while calculating tumor burden aids in assessing high-risk patients suitable for SIRT, CTA is not feasible for evaluating liver perfusion volume.

Cone Beam CT (CBCT) provides high-quality cross-sectional images and is widely used in liver, vascular, and tumor imaging. Unlike CTA, CBCT achieves more precise staining effects in the tumor area by directly injecting contrast agents into the tumor-feeding arteries. CBCT images offer more accurate indication of enhanced tumor areas, making them valuable for tumor interventional surgery planning and treatment implementation. This technology has demonstrated improved liver tumor detection and prediction of ^90^Y microsphere distribution (16–21). It is particularly beneficial for procedures such as transarterial chemoembolization (TACE) and SIRT in treating HCC (21). Moreover, variations in blood supply from the hepatic artery often introduce uncertainties in blood perfusion patterns. For example, the right posterior hepatic artery may occasionally supply the right anterior lobe, and the middle hepatic artery may intermittently supply the right lobe. Given these uncertainties, CBCT images provide accurate analysis (1, 5, 11, 12, 21, 22). However, arterial phase imaging has its limitations. Therefore, this study employs dual CBCT images for comparative analysis.

This study aims to explore the feasibility of using dual CBCT images as a substitute for preoperative CTA data in calculating radiation dosage based on liver lobe and tumor volume in ^90^Y-SIRT treatment.

## 2 Materials and methods

### 2.1 Patient cohort

This monocentric retrospective study was approved by a local ethics and institutional review board committee. This observational study analyzed 120 patients with unresectable primary or metastatic liver tumors eligible for SIRT from October 2022 to July 2024. Prior to angiography, ^99m^Tc-MAA injection, and dual CBCT planning, each patient underwent contrast-enhanced four-phase CTA of the liver. Rigorous exclusion criteria were applied to ensure the cohort homogeneity and reliability. The study focused on unilateral liver perfusion, either in the right or left lobes, emphasizing the impact of different imaging modalities on volume assessment to minimize measurement errors. Seventy-four patients were excluded due to non-necessity of hepatic segmentectomy, 14 due to cancer spanning both liver lobes, 4 due to large lesions (over 20 cm), and 12 due to having more than four lesions. Additionally, 13 participants were excluded due to lack of postoperative imaging within two months. Patients who failed to undergo CBCT scanning or underwent planning angiography performed more than 3 weeks after the liver CTA examination were also excluded.

Ultimately, 27 patients (mean age: 58.0 years; 24 males, 3 females) were included. The demographic and clinical characteristics were detailed in **Table 1**. The average time interval between liver CTA and ^90^Y-SIRT was 10.68 days (range: 6-17, median:11), while between dual CBCT (same date as mapping) and SIRT was 7.29 days (range: 5-9, median:7). Baseline diagnosed included HCC in 20 patients and metastases/intrahepatic cholangiocarcinoma (ICC) in 7 patients. Among these, 10 cases targeted the left liver lobe, and 17 targeted the right lobe. The treatment area comprised a single tumor in 6 patients and multiple tumors in 21 patients.

**Table 1:** Patient demographics and clinical data.

**Number of patients**	27
**Gender (male-to-female)**	24:3
**Age (mean, range)** **Albumin (mean, range)**	58, (35-75) 36.5, (3.9-62.0) g/dL
Tumor type (pcs)	
HCC Metastases/ICC	20 7
Number of tumors	
single multiplicity	6 21
BCLC grade(total 20 patients)	
II A II B III A III B	1 4 8 7
Target tumor location(pcs)	
Right Liver Left Liver	17 10

### 2.2 Liver multi-phase CTA scan

A multi-slice spiral CTA was used for the liver four-phase scan. The patient lay supine with the scanning range extending from the diaphragmatic dome to the iliac crest. Parameters were as follows: tube voltage of 120 kV, automatic tube current, pitch ratio of 0.992:1, slice thickness of 5 mm, and interlayer spacing of 5 mm. A contrast agent (iodixanol 350 mg/ml) is injected via a high-pressure injector through the median cubital vein at 3 ml/s. Arterial, portal venous, venous, and delayed phase scans were acquired at 29s, 45s, 60s, and 180s post-injection, respectively.

### 2.3 ^90^Y-SIRT treatment planning

All patients in this study were recently diagnosed with unresectable liver tumors, aligning with the SIRT consensus for ^90^Y microspheres in Asian hepatocellular carcinoma (7). A multidisciplinary oncology committee, including minimally invasive interventional, radiology, nuclear medicine, hepatobiliary surgery, and medical oncology specialists, individually reviewed each patient’s history, clinical status, and imaging results to discuss therapeutic strategies. The committee unanimously agreed on SIRT as the chosen treatment for all study participants.

**Figure 1:**
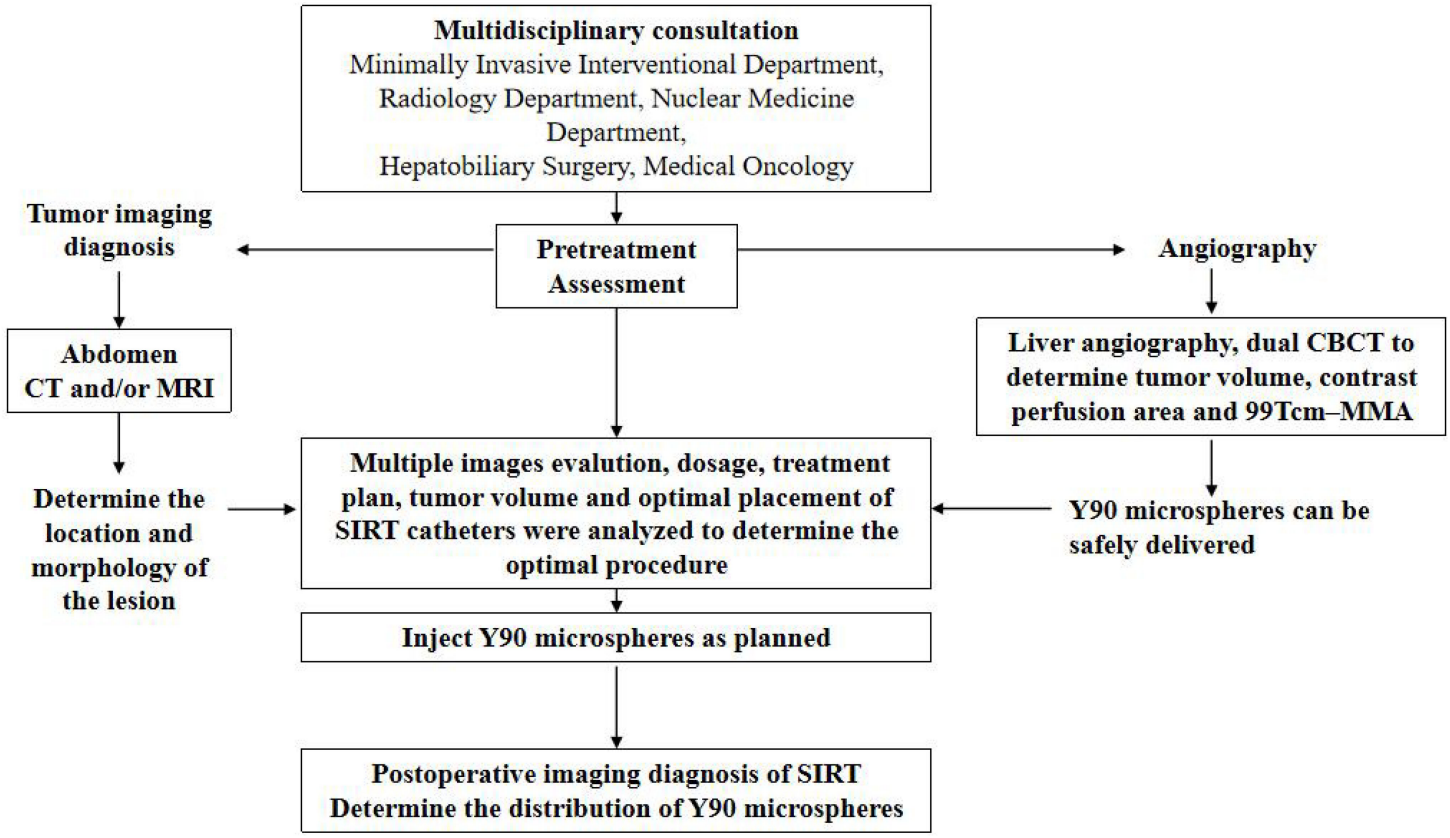
The flowchart of 90Y-SIRT procedure for liver cancer treatment.

Experienced interventional radiologists performed angiography and SIRT treatment planning, adhering to our standard protocol (**Figure 1**). Preoperative CTA images were reviewed to identify any unusual arterial anatomy supplying the liver. The right-sided femoral artery route was used in all cases. A celiac artery or superior mesenteric arteriogram was then performed using a 5F Terumo RH catheter to assess the vascular supply to the liver and target lesions. Hyperselective conventional angiography was conducted using Tokai 2.2F or Tokai 1.8F microcatheters.

During the planning angiography, dual CBCT (Philips UNIQ FD20C, Philips Medical, Netherlands) was performed to identify feeding arteries and assess perfusion to the liver tumor, ensuring accurate tumor enhancement identification. For patients with right lobe tumors, the catheter tip was strategically placed to achieve a single perfusion volume or positioned in the right anterior and right posterior arteries to obtain two separate perfusion volumes. The sum of these two volumes defined the perfusion range of the entire right liver lobe. If the left lobe or middle lobe supplied blood to the right lobe, their volumes were included in the total volume. A similar approach was applied for the left liver lobe. Finally, ^99m^Tc-MAA simulation surgery was performed at the anticipated SIRT site.

### 2.4 Dual CBCT technology

**Figure 2:**
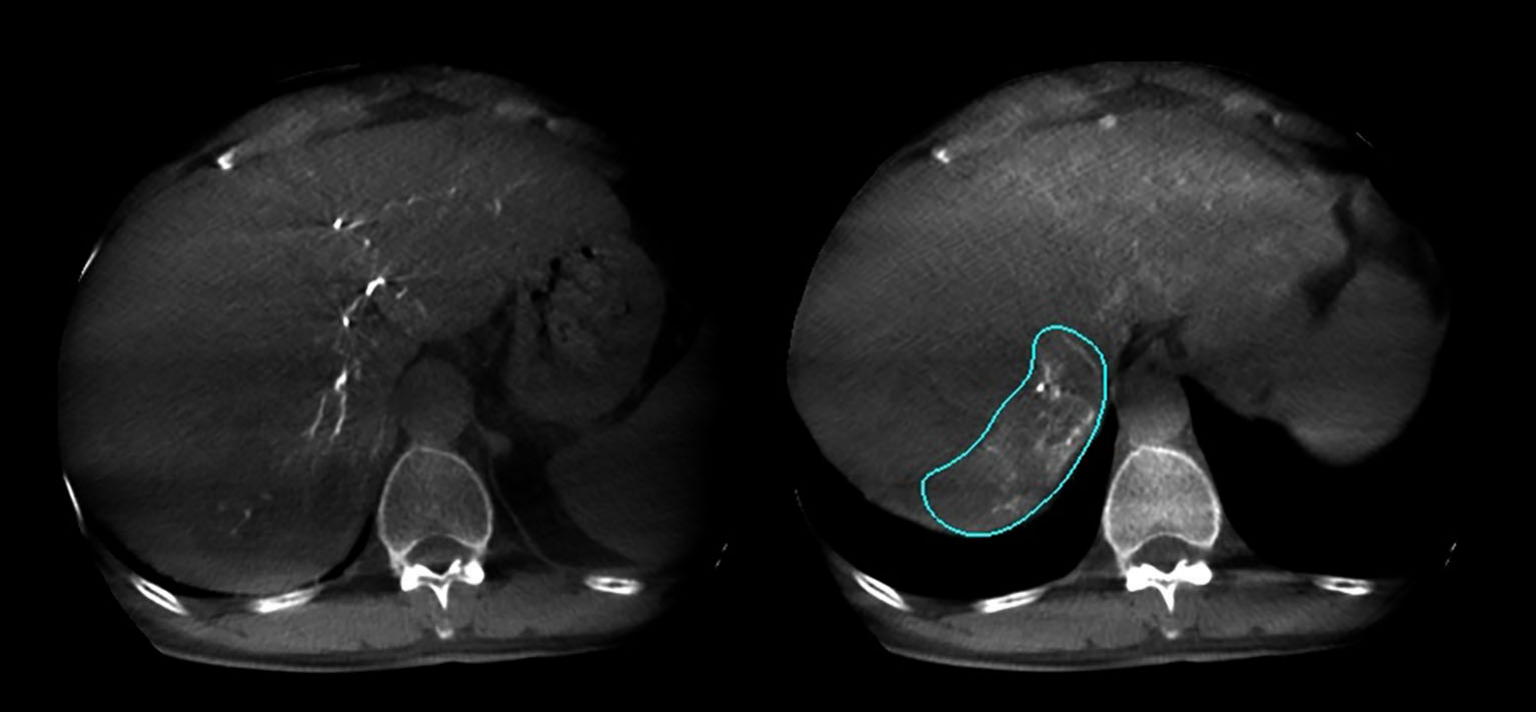
Arterial phase and delayed phase contrast-enhanced dual CBCT images acquired after injection into the tumor-feeding artery. The tumor area can be assessed based on the delayed phase image on the right.

**Figure 3:**
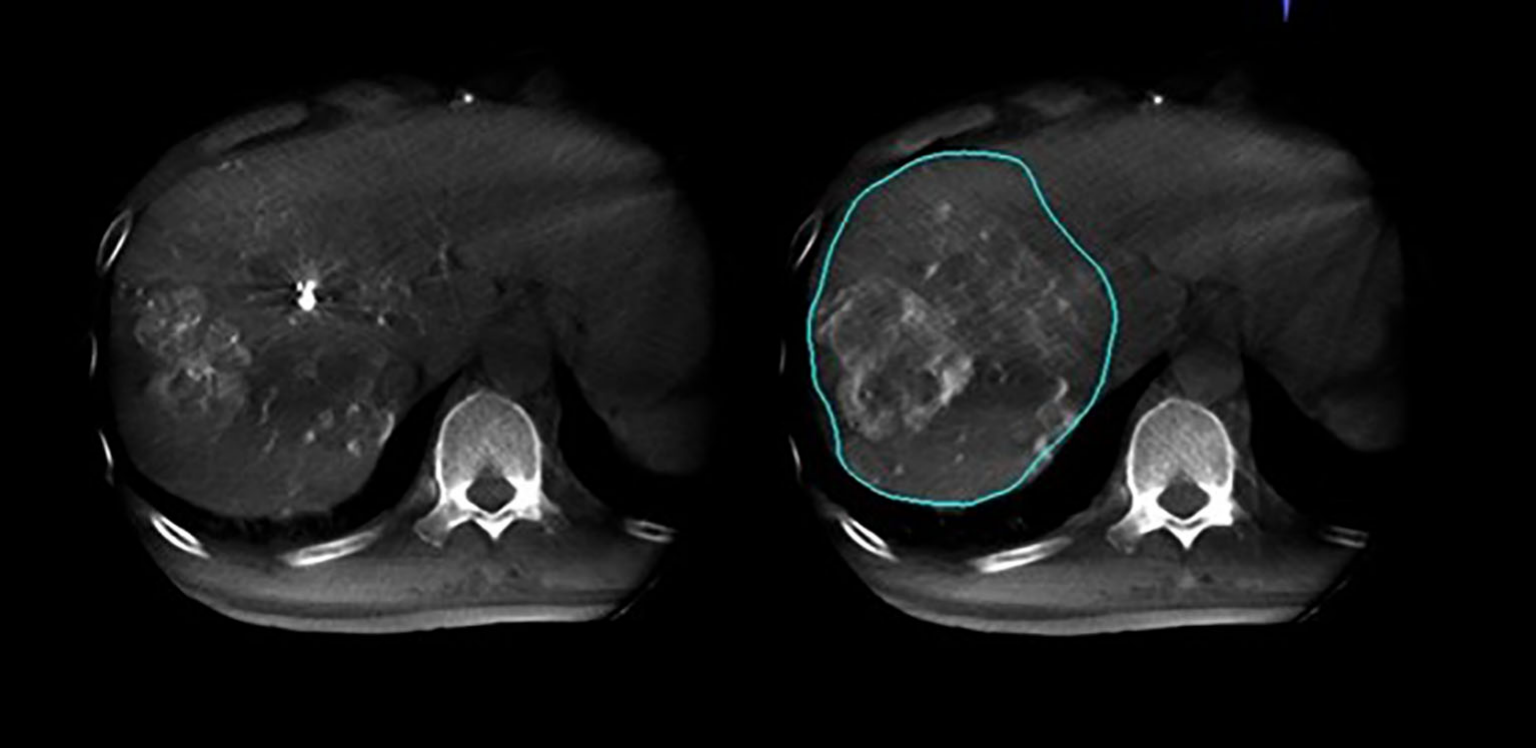
Arterial phase and delayed phase contrast-enhanced dual CBCT images acquired after injection into the segmental right hepatic lobe. The perfusion area can be assessed based on the delayed phase image on the right.

In this study, 27 patients underwent liver DSA and dual CBCT scans prior to SIRT treatment to evaluate intrahepatic lesions, identify tumor-feeding vessels, ensure tumor enhancement, and exclude blood supply from collateral vessels. The Philips UNIQ FD20C (Philips Healthcare, Netherlands) was used. The dual CBCT was automatically triggered for image acquisition during the arterial and delayed phases. The flat panel detector rotated from -120° to +120°, enabling a comprehensive liver parenchyma scan. X-ray projections were acquired at 60 frames per second over 240°. The X-ray exposure was set to 120 kV, with a detector size of 48 cm and a pixel size of 4x4. Dual CBCT was performed during proper hepatic artery angiography by injecting 18 mL of contrast medium (Visipaque 320 mg I/ml) at a rate of 1.5 mL/s to evaluate total liver volume, right and left lobe volumes, and overall tumor burden. The first scan was initiated 4 seconds after the start of contrast injection, followed by a second scan commencing 8 seconds after the completion of the first, with each scan lasting 8 seconds. Subsequently, superselective DSA was conducted to assess the perfusion volume of specific hepatic arterial branches and the corresponding tumor volumes. For these injections, the contrast infusion rate was typically set at one-tenth of that used for conventional angiography. The total injection duration was defined as the time required for parenchymal opacification plus the rotation time of the imaging system, thereby ensuring that image acquisition occurred when the tumor region and its feeding arteries reached peak contrast enhancement. The total contrast volume administered was calculated as the product of the selected injection rate and injection duration. Contrast-enhanced dual CBCT data allowed precise measurement of the target liver lobe volume and tumor burden via a 3D segmentation tool. Arterial and delayed phase CBCT images enabled tumor area assessment, particularly during the delayed phase image (**Figure 2**). Injection into the segmental right hepatic artery provided detailed tumor perfusion area assessment. Both arterial and delayed phase images, especially the delayed phase (**Figure 3**), offered precise information on tumor perfusion.

### 2.5 Liver lobe and tumor burden volume analysis

The Philips Interventional Workstation was used with dual CBCT for segmentation and volume calculation. The volume of each liver lobe was measured from angiographic images, while tumor burden was assessed based on the size of high-density tumors. Target liver lobe volume and tumor burden were calculated using arterial late phase and portal venous phase of the liver CTA. Two experienced interventional radiologists (over 10 years of experience) independently measured liver lobe and tumor volumes from both CTA and dual CBCT images in random order. They analyzed data from 12,960 dual CBCT images across 27 patients.

### 2.6 Nuclear medicine and ^90^Y radioembolization

In this study, preoperative validation of the ^90^Y procedure was supported by two physicians from the hospital’s Nuclear Medicine department. This included calculating the lung shunt fraction (LSF) for ^90^Y radiation, determining the dose ratio for tumor versus normal tissue, and calculating the radioactivity for ^90^Y microspheres injection. At our institution, ^90^Y dosages were calculated using the Medical Internal Radiation Dose (MIRD) equation based on liver lobe mass, which was derived from the measured liver lobe volume. The ^90^Y-SIRT dosage was calculated based on preoperative tumor and perfusion volumes, following the manufacturer’s guidelines for dosing ^90^Y resin microspheres. These guidelines use a nonsegmental MIRD approach to target a dose of 120 Gy. The liver tumor burden was calculated as the percentage volume sum of either a single tumor or the largest three tumors within the target liver lobe. The average LSF value was 10.76%, with a range of 3.37% to 32.50%.


$$Activity\;required\;(GBq)=\frac{Dose_{normal}}{50}*(M_{normal}+TNR*M_{tumor})*\frac{1}{1-LSF}$$

### 2.7 Statistical analysis

Statistical analysis was conducted using SPSS 27.0 software. Descriptive statistics were calculated for the patients’ average data. The Shapiro-Wilk test confirmed that liver and tumor volumes did not follow a normal distribution. The paired Wilcoxon test was used to analyze differences in liver lobe and tumor volumes, as well as ^90^Y microsphere dosage, between the two imaging techniques. A p-value less than 0.05 was considered statistically significant.

## 3 Results

### 3.1 Measurement of tumor volume and perfusion volume

Using CTA and dual CBCT images, target liver lobes and tumors were clearly visible without assessment limitations or artifacts. The liver lobe volume(LLV) from dual CBCT (LLVcbct) was 2083.88 ± 744.64 cm³, while from CTA (LLVcta) was 2187.86 ± 807.28 cm³. A significant difference in LLV was observed between the imaging modalities (p = 0.024). The mean tumor volume(TV) in the liver (TVcta) was 542.09 ± 547.24 cm³ on CTA and (TVcbct) 516.31 ± 482.55 cm³ on dual CBCT. Liver perfusion volume(LPV) assessed via ^99m^Tc-MAA mapping (LPVmma) was 983.11 ± 658.92 cm³, compared to (LPVcbct) 957.61 ± 631.49 cm³ by dual CBCT (**Table 2**). No significant differences in liver tumor and perfusion volume measurements were found between the two modalities (p = 0.792 and 0.084, respectively).

**Table 2:** Assessment of treatment volume.

Assessment parameters		p value
**Liver lobe volume** (mean±SD)		p= 0.024
LLVcta:CTA	2187.86 ±807.28	
LLVcbct:Dual CBCT	2083.88 ±744.64	
**Tumor volume** (mean±SD)		p= 0.792
TVcta:CTA	542.09± 547.24	
TVcbct:Dual CBCT	516.31± 482.55	
**Liver perfusion volume** (mean±SD)		p= 0.084
LPVmma:MMA	983.11±658.92	
LPVcbct:Dual CBCT	957.61±631.49	

### 3.2 ^90^Y dosage

Statistical analysis revealed an average calculated ^90^Y dosage based on tumor volume and liver perfusion measured by dual CBCT of 1.819 ± 1.241 GBq (range: 0.26 - 4.52). In comparison, using tumor volume from dual CBCT and liver perfusion measured by ^99m^Tc-MAA yielded a ^90^Y dosage was of 1.806 ± 1.240 GBq (range: 0.25 - 4.39). The mean clinically administered radioactivity was 1.805 ± 1.236 GBq (range: 0.30 - 4.39). No significant differences were found between the ^90^Y dosage calculations from dual CBCT and ^99m^Tc-MAA mapping (p = 0.555) as shown in **Table 3**. A p-value less than 0.05 was considered indicative of a significant difference. One patient received a calculated dose below 0.30 GBq; however, due to a minimum prescribed dose of 0.30 GBq, the actual administered dose was slightly higher.

**Table 3:** Assessment of ^90^Y dosage by different volume results from modalities images (T, Tumor; P, Perfusion).

^90^Y dose calculation results	Value (mean±SD)	p value
TVcbct & LPVcbct vs. TVcbct & LPVmma	1.819±1.241 1.806±1.240	p=0.555
TVcbct & LPVcbct vs. Radioactivity	1.819±1.241 1.805±1.236	p=0.525
TVcbct & LPVmma vs. Radioactivity	1.806±1.241 1.805±1.236	p=0.739

### 3.3 Enhanced tumor feeder arteries identification with dual CBCT

**Figure 4:**
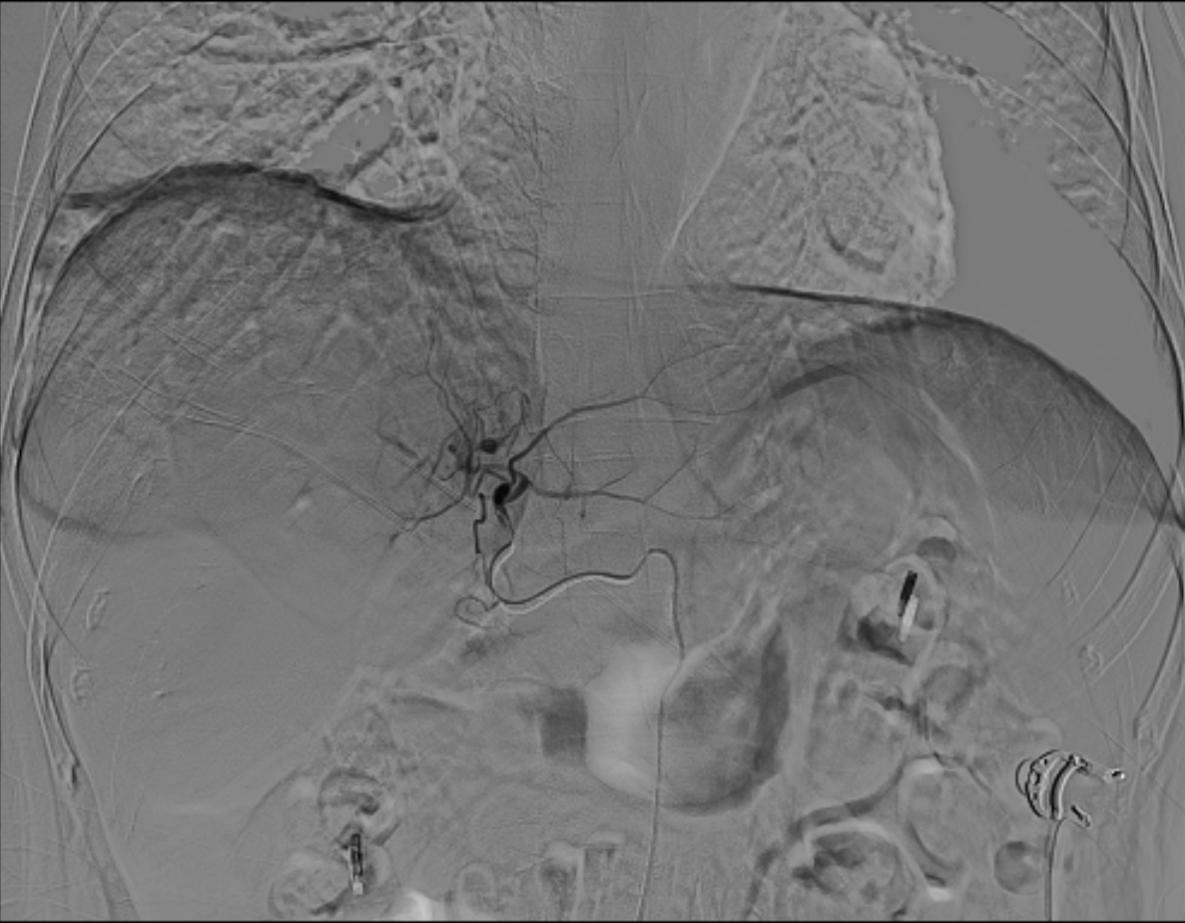
Conventional 2D angiography shows overlapping normal hepatic arterial branches and tumor-feeding arteries, making distinction challenging.

**Figure 5:**
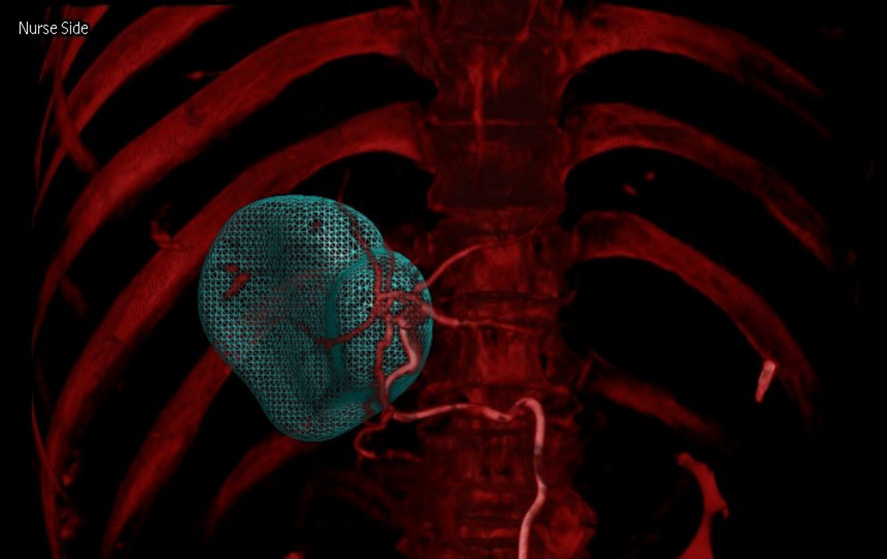
Combining arterial and venous phase CBCT imaging (dual CBCT-overlay image) enables rapid and accurate identification of tumor-supplying arteries, facilitating more precise ^90^Y therapy delivery.

As illustrated in **Figure 4** and **Figure 5**, conventional 2D angiography often present overlapping normal hepatic arterial branches and tumor-feeding arteries, making their distinction challenging. In contrast, dual CBCT enabled rapid and accurate identification of tumor-feeding arteries, enhancing the precision of ^90^Y therapy delivery. This technology successfully identified tumor-feeding arteries in all 27 cases, achieving a 100% success rate.

### 3.4 Optimizing tumor-feeding artery identification and catheter insertion

**Figure 6:**
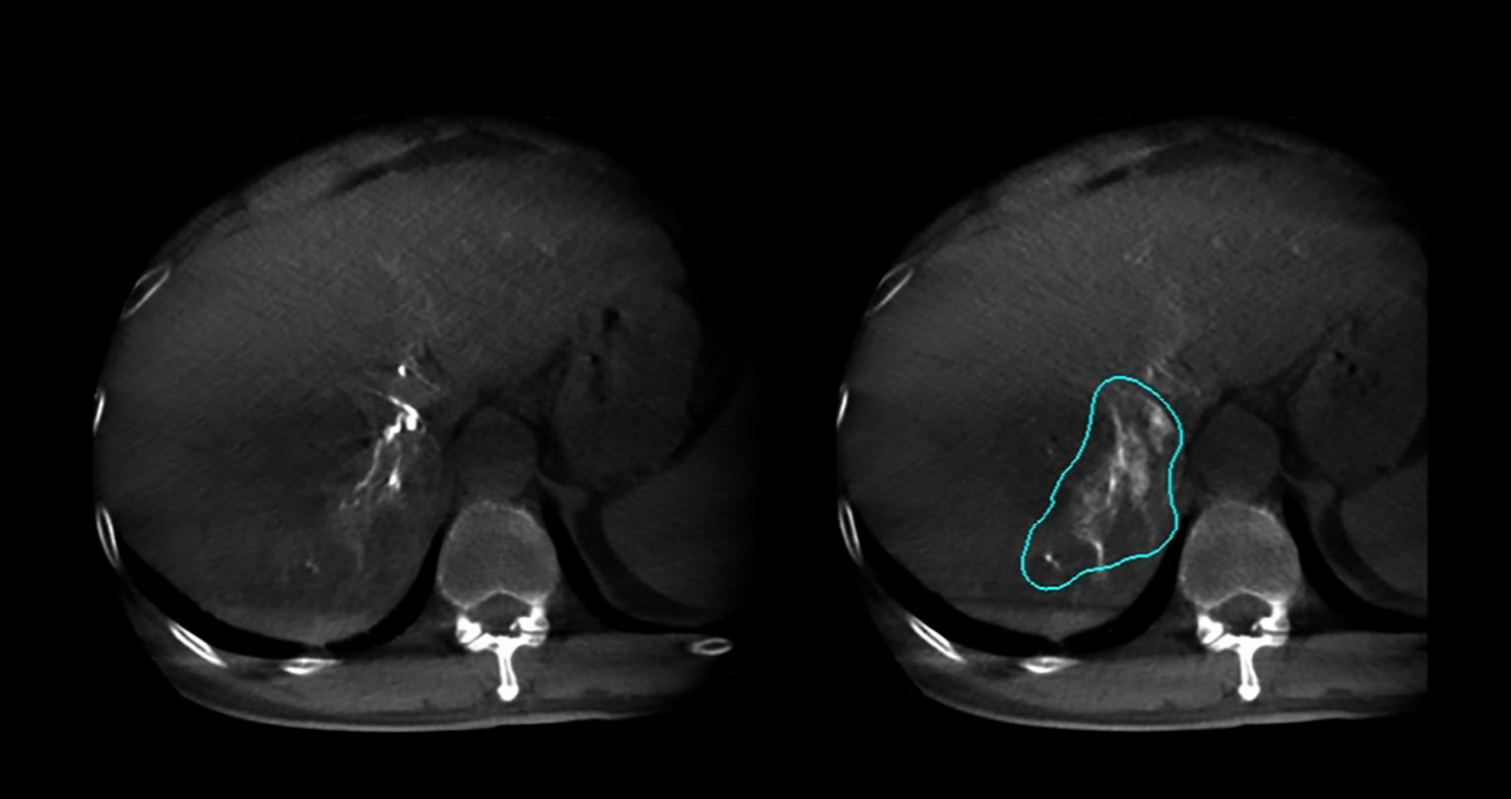
Dual CBCT images from the same cross-section were used to create a three-dimensional reconstruction model of the tumor.

**Figure 7:**
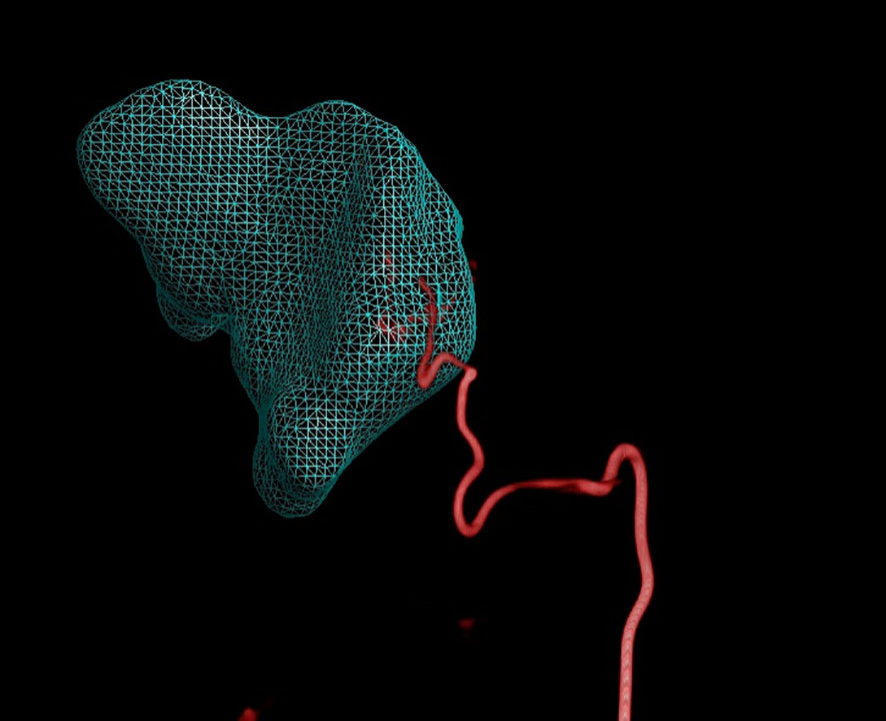
Dual CBCT used to create a 3D reconstruction model of its blood supply vessels (viewed from a left anterior oblique position) after successful super-selection.

**Figure 8:**
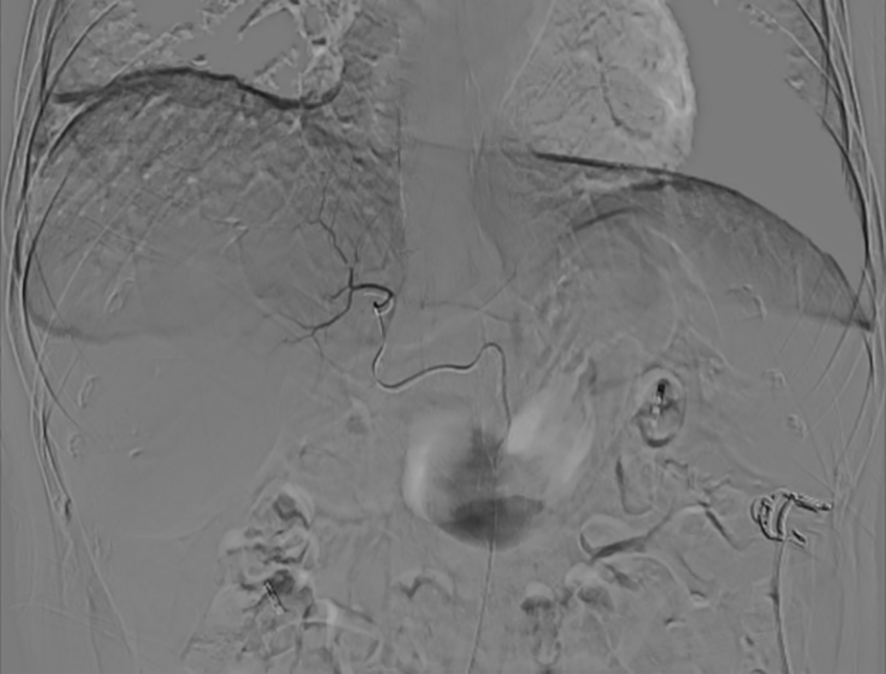
Confirmation with arteriography performed after super-selection.

This study utilized dual CBCT technology to precisely locate tumors and their feeding vessels. **Figure 6** and **Figure 7** showed dual CBCT from the same cross-section obtained after super-selection, used to construct a 3D reconstruction model of the tumor and its blood supply vessels. The 3D model, viewed from a left anterior oblique position, clearly displays the spatial relationships between the tumor and its vessels. This detailed reconstruction significantly enhanced the precision and effectiveness of subsequent interventional procedures. **Figure 8** illustrated the confirmation process conducted via arteriography after super-selection, verifying vascular localization and providing reliable data for subsequent operations.

**Figure 9:**
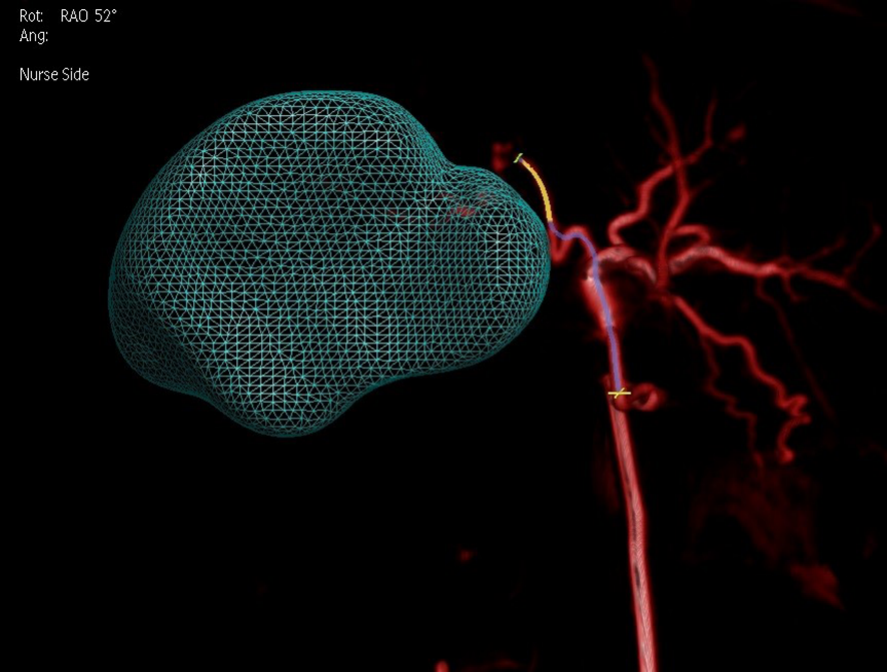
Adjustment of the angle (52 degrees right anterior oblique in the fused model) on a 3D model based on two-phase image reconstruction aids in better identification of tumor-feeding arteries and precise localization of their entry points for catheter insertion.

**Figure 10:**
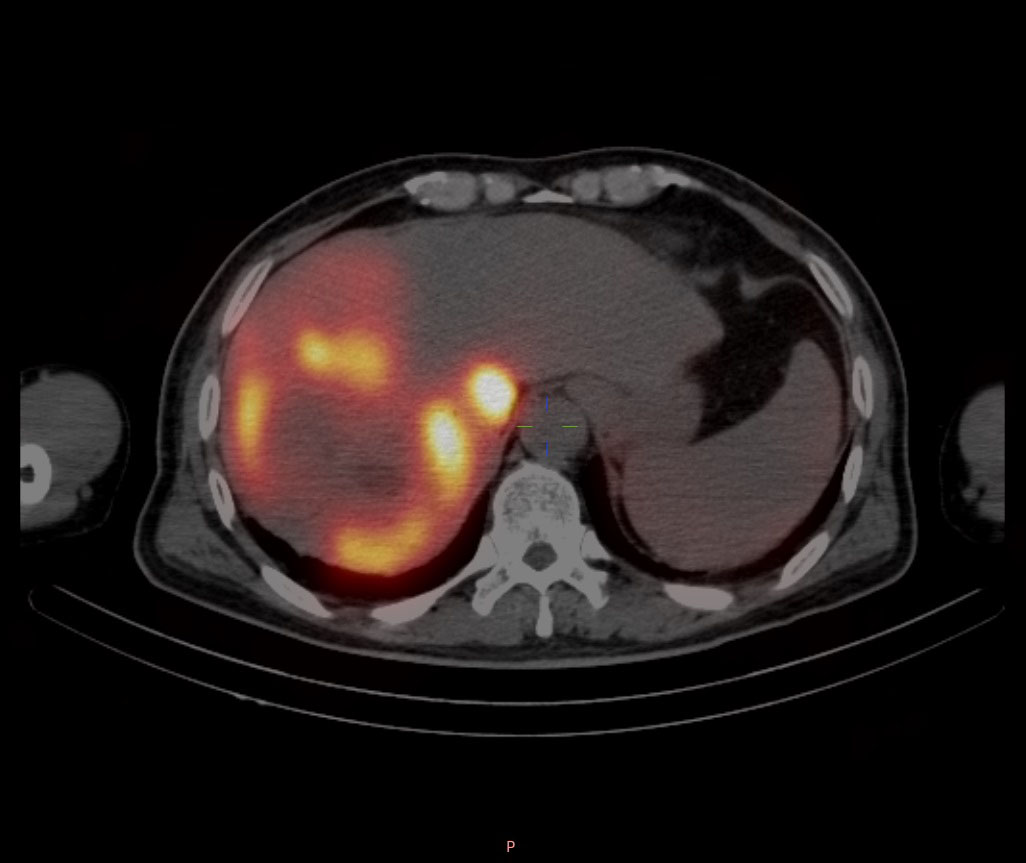
Nuclear medicine verification performed after completion of the 90Y-SIRT procedure.

By adjusting the angle, better identification of tumor-feeding arteries was achieved, assisting operators in accurately locating their entry points for precise catheter insertion. The angle was set to 52° right anterior oblique in the fused model, as shown in **Figure 9**. Post-^90^Y-SIRT, nuclear medicine verification was performed, as shown in **Figure 10**.

### 3.5 Radiation dose analysis of dual CBCT in ^90^Y therapy

This study analyzed the average Dose Area Product (DAP) and Air Kerma (AK) among different patients. Due to the manual recording of radiation dose tables using the FD20 DSA system, only 22 out of 27 patient’s dose tables were collected for analysis. The results, serving as reference value, indicated that the dual CBCT technology in ^90^Y-SIRT doesn’t significantly increase radiation dose. The average DAP was 15.26 ± 3.27 Gy*cm², and the average AK was 44.41 ± 7.92 mGy.

## 4 Discussion

This preliminary study underscored the potential of dual CBCT as a reliable tool for dose calculation in SIRT using yttrium-90 microspheres. Our findings systematically evaluated the advantages of dual CBCT in liver tumor treatment. It not only demonstrated strong consistency with preoperative CTA in providing accurate tumor volume measurement but also calculated perfusion territories, enabling more precise ^90^Y dose calculation. In addition, dual CBCT overcame the limitations of conventional DSA by offering 3D visualization of tumor–vessel relationships, which improved feeder identification, shortened procedure time, and enhanced treatment success.

### 4.1 Technical development and preoperative evaluation

Over the past two decades, CBCT technology has significantly improved the contrast-to-noise ratio (CNR) and addressed anatomical limitations in covering the entire liver. Dual CBCT has proven to offer precision comparable to preoperative intravenous contrast-enhanced dual dynamic multi-detector CTA (18). In this study, dual CBCT yielded tumor volume measurements consistent with those from CTA. Specifically, the tumor volumes measured by CTA (542.09 ± 547.24 cm³) and dual CBCT (516.31 ± 482.55 cm³) exhibited no significant difference (p = 0.792). Similarly, dual CBCT provided liver perfusion volumes measurements comparable to ^99m^Tc-MAA mapping 983.11 ± 658.92 cm³ *vs*. 957.61 ± 631.49 cm³, p = 0.084). These findings indicated that dual CBCT can effectively approximate the volumetric measurements necessary for accurate dose calculation in SIRT.

Notably, both tumor and liver volumes measured using dual CBCT were systematically smaller than those obtained from CTA. The liver volume measured from dual CBCT (2083.88 ± 744.64 cm³) differed significantly from that by CTA (2187.86 ± 807.28 cm³, p = 0.024). This discrepancy may be attributed to two main factors. First, differences in contrast agent administration and delivery played a key role. CTA scans used peripheral venous injections, enhancing both the tumor and liver surrounding parenchyma, potentially diluting contrast concentration within the tumor. Conversely, dual CBCT uses direct intra-arterial injections, resulting in more precise tumor staining and a more accurate delineation of enhancing tumor area. Second, the methods of volume measurement differed between modalities. The CTA-based liver volume was semi-automatically segmented using a CT post-processing workstation, whereas tumor volumes and perfusion territories from both CTA and CBCT were manually contoured on the angiographic system’s workstation. This methodological difference may also contribute to the inconsistent liver volume results. Furthermore, previous findings by Seth I. Stein et al. (23) have demonstrated that factors such as proximal versus distal microcatheter positioning, arterial anatomical variations, and tumor proximity to the segmental vascular territory can further contribute to differences between CBCT- and CT/MRI-based volumetry.

In this study, dual CBCT achieved a 100% display rate for target liver lobe segments, tumors, and perfusion volume without noticeable artifacts. These results underscored dual CBCT’s capacity for accurate volume assessment in SIRT, paving the way for more precise and effective radiation therapy.

### 4.2 ^90^Y dose accurate calculation

The ^90^Y dosage calculated using tumor volume and perfusion volume measurements from dual CBCT was 1.819 ± 1.241 GBq, which closely aligned with the dosage (1.806 ± 1.240 GBq) derived from tumor volume assessed from dual CBCT and perfusion volume measured by ^99m^Tc-MAA (p = 0.555). Additionally, the mean clinically administered dosage was 1.805 ± 1.236 GBq, showing no significant difference from the dosage calculated by dual CBCT (p = 0.525). This indicated that dual CBCT provided a dose estimation consistent with clinical practice. Moreover, the ^90^Y resin microsphere dosage calculated by dual CBCT and perfusion mapping exhibited no significant differences (p = 0.739).

### 4.3 Vascular visualization and accurate catheterization

Dual CBCT technology achieved a 100% accuracy in tumor-feeding vessels localization, which significantly enhanced the surgeons’ ability to identify and manipulate vessels. This improvement boosted surgical success rates and reduced procedure duration. By accurately identifying vessel openings, dual CBCT facilitated precise super-selection catheterization, enhancing treatment precision and advancing precision medicine.

### 4.4 Time interval for pre-SIRT liver imaging examinations

In this study, 27 patients underwent dual CBCT an average of 7.29 days (range: 5-9, median: 7) before ^90^Y treatment, while CTA examinations were performed 10.68 days (range: 6-17, median:11) prior. Theoretically, the interval between ^90^Y-SIRT and CBCT could be reduced to 2 days, mainly due to ^99m^Tc metabolism. However, current ^90^Y drugs imports from Singapore incur a week-long transportation time to Chinese hospitals. Planned ^90^Y drug production in China by 2025 may shorten delivery time.

### 4.5 Economic and clinical operational advantages

In addition to its technical performance, the integration of dual CBCT into the SIRT workflow demonstrated economic and clinical operational benefits. Specifically, the dual CBCT protocol, which incorporates an arterial phase acquisition complementing the conventional delayed-phase scan, did not substantially prolong procedural duration. More importantly, by improving the identification of feeding arteries and reducing the need for repeated angiography acquisitions, the protocol potentially enhanced overall workflow efficiency and contributed to a reduction in total procedure time. Consequently, this approach enabled more comprehensive imaging assessment without modifying the existing clinical workflow.

Regarding economic impact, CBCT is not categorized as a separately billable service within the current reimbursement framework. Furthermore, since the contrast agent required was incorporated into the existing surgical material budget, the incremental cost remained minimal. Operational workflow, the implementation did not increase procedural complexity or necessitate additional staffing resource, while equipment requirements remained fully consistent with the conventional single-phase CBCT workflow. From a safety perspective, the additional arterial phase acquisition required only approximately 8 seconds, and the associated radiation exposure remained within established safety range.

In summary, the incorporation of dual CBCT into the SIRT workflow represents a cost-effective technical advancement, improving tumor characterization and procedural planning without substantially increasing operational costs, ultimately achieving an optimal integration of clinical efficacy and economic efficiency.

## 5 Limitation

This feasibility study, a single-center, small-sample observational study involving 27 patients, may limit the generalizability of the results to a broader population undergoing ^90^Y-SIRT. Evaluating reproducibility among observers may present challenges due to varying levels of experience and the learning curve associated with new segmentation techniques. The study is limited by the absence of follow-up data on tumor response and survival, which restricts evaluation of the clinical impact of imaging differences. Future studies should incorporate longitudinal follow-up to assess treatment outcomes and validate the clinical significance of dual CBCT measurements.

## 6 Conclusion

This study preliminarily demonstrated that dual CBCT provided comparable accuracy in measuring liver tumor volumes and assessing perfusion volumes compared to traditional CTA and ^99m^Tc-MAA mapping. Additionally, the ^90^Y doses derived from dual CBCT closely match those used in clinical practice, highlighting its potential for accurate clinical dose estimation. Prospective multicenter studies with long-term follow-up are needed to validate these findings and determine the impact of dual CBCT on patient outcomes.

## Data Availability

The original contributions presented in the study are included in the article/supplementary material. Further inquiries can be directed to the corresponding authors.
